# Bone marrow involvement identifies a subgroup of advanced Ewing sarcoma patients with fatal outcome irrespective of therapy in contrast to curable patients with multiple bone metastases but unaffected marrow

**DOI:** 10.18632/oncotarget.10938

**Published:** 2016-07-29

**Authors:** Uwe Thiel, Angela Wawer, Irene von Luettichau, Hans-Ulrich Bender, Franziska Blaeschke, Thomas G.P. Grunewald, Marc Steinborn, Barbara Röper, Halvard Bonig, Thomas Klingebiel, Peter Bader, Ewa Koscielniak, Michael Paulussen, Uta Dirksen, Heribert Juergens, Hans-Jochem Kolb, Stefan E.G. Burdach

**Affiliations:** ^1^ Department of Pediatrics and Pediatric Oncology Center, Kinderklinik München Schwabing, Städtisches Klinikum München und Klinikum rechts der Isar, Wilhelm Sander Sarcoma Unit, Klinikum rechts der Isar, Technische Universität München, Munich, Germany; ^2^ Laboratory for Pediatric Sarcoma Biology, Institute of Pathology, LMU, Munich, Germany; ^3^ Department of Radiology, Klinikum Schwabing, Städtisches Klinikum München, Munich, Germany; ^4^ Department of Radiation Oncology, Klinikum rechts der Isar, Technische Universität München, Munich, Germany; ^5^ Department of Pediatric Hematology and Oncology, Universitätsklinikum Frankfurt, Frankfurt, Germany; ^6^ Department of Transfusion Medicine and Immunohematology, Universitätsklinikum Frankfurt, Frankfurt, Germany; ^7^ Department of Pediatric Oncology, Hematology and Immunology, Olgahospital, Klinikum Stuttgart, Stuttgart, Germany; ^8^ Vestische Kinder- und Jugendklinik, Datteln, Universität Witten/Herdecke, Datteln, Germany; ^9^ Department of Pediatric Hematology and Oncology, Universitätsklinikum Münster, Münster, Germany; ^10^ Munich Comprehensive Cancer Center, München, Germany

**Keywords:** Ewing sarcoma, bone metastasis, bone marrow metastasis, allogeneic stem cell transplantation, high-dose chemotherapy

## Abstract

**Purpose:**

Advanced Ewing sarcomas have poor prognosis. They are defined by early relapse (<24 months after diagnosis) and/or by metastasis to multiple bones or bone marrow (BM). We analyzed risk factors, toxicity and survival in advanced Ewing sarcoma patients treated with the MetaEICESS vs. EICESS92 protocols.

**Design:**

Of 44 patients, 18 patients were enrolled into two subsequent MetaEICESS protocols between 1992 and 2014, and compared to outcomes of 26 advanced Ewing sarcoma patients treated with EICESS 1992 between 1992 and 1996. MetaEICESS 1992 consisted of induction chemotherapy, whole body imaging directed radiotherapy to the primary tumor and metastases, tandem high-dose chemotherapy and autologous rescue. In MetaEICESS 2007 this treatment was complemented by allogeneic stem cell transplantation. EICESS 1992 comprised induction chemotherapy, local therapy to the primary tumor only followed by consolidation chemotherapy.

**Results:**

In MetaEICESS 8/18 patients survived in complete remission vs. 2/26 in EICESS 1992 (p<0.05). Survival did not differ between MetaEICESS 2007 and MetaEICESS 1992. Three MetaEICESS patients died of complications, all in MetaEICESS 1992. After exclusion of patients succumbing to treatment related complications (n=3), 7/10 patients survived without BM involvement, in contrast to 0/5 patients with BM involvement. This was confirmed in a multivariate analysis. There was no correlation between BM involvement and the number of metastases at diagnosis.

**Conclusion:**

The MetaEICESS protocols yield long-term disease-free survival in patients with advanced Ewing sarcoma. Allogeneic stem cell transplantation was not associated with increased death of complications. Bone marrow involvement is a risk factor distinct from multiple bone metastases.

## INTRODUCTION

Ewing sarcomas are defined by t(11;22)(q24;q12) derived EWS/ETS fusion oncogenes [1]. They occur in both bone and soft tissue [2]. Advanced Ewing sarcoma comprises early relapse or metastatic to multiple bones (i.e. more than one) or bone marrow (BM) [3, 4]. Overall survival of advanced Ewing sarcoma after ten years is ≤10% [4–6], warranting the study of intensified treatment modalities [6–19]. Moreover, the distinct contribution of BM involvement vs. multiple bone metastases to the dismal prognosis is not defined.

In the MetaEICESS 1992 protocol we used induction chemotherapy, whole-body MRI (whole-body MRI) and PET based primary and metastatic tumor irradiation combined with autologous stem cell rescue, followed by tandem high-dose chemotherapy with additional autoologous rescue as consolidation [4]. In the prospective MetaEICESS 2007 study, this sequence was complemented by reduced intensity conditioning and allogeneic stem cell transplantation. Results of disease-free survival of both MetaEICESS protocols were compared with advanced Ewing sarcoma patients who received the EICESS 1992 regimen with induction and consolidation chemotherapy and primary tumor radiation only. Furthermore, results of the MetaEICESS 1992 [4] and the EICESS 1992 groups [22] previously published are updated here.

### Definitions

Advanced Ewing sarcoma was defined by early relapse (<24 months after diagnosis) and/or by metastatic disease to multiple bones or BM. BM involvement was defined as cytological detection of tumor cells in the BM aspirate. Death of disease comprised any death directly related to disease progression or relapse. Complete remission was defined as lack of tumor evidence. Death of complication was any death attributable to therapy. In this report, disease-free survival was defined as the interval between the date of diagnosis that prompted respective protocol admission until the occurrence of any local or metastatic tumor evidence (or death of complications when indicated). In MetaEICESS 2007 patients we determined a second disease-free interval as well as overall survival which was was defined as the time period from the last allogeneic stem cell transplantation until first relapse, death or last follow-up (compare Supplementary Figure S1).

## PATIENTS AND METHODS

44 patients were assessed in this study: 18 patients enrolled in the two subsequent single-center MetaEICESS 1992 (registered from 1995 to 2000) and 2007 protocols (registered from 2007 to 2014) were compared to 26 controls (EICESS 1992; registered from 1992 to 1996). Eligibility criterion was diagnosis of advanced Ewing sarcoma. The cut off of ≥ 2 bone metastases and/or early relapse (relapse<24 months after diagnosis) was identical to previous studies [4, 23]. Patients were only treated with the according protocol, respectively, and did not switch groups during treatment course. All patients and their legal guardians signed informed consent prior to therapy. Protocol treatment was applied after approval by the institutional review boards according to the precepts established by the Helsinki Conference Declaration.

### EICESS 1992

In the EICESS group 26 patients with multiple bone metastases at diagnosis (aged 6–37 years; median: 17 years) were registered from 1992 to 1996 and treated according to the EICESS 92 protocol for advanced Ewing sarcoma, as previously described. In order to prevent a selection bias in favor of MetaEICESS and to warrant comparability to patients treated with the MetaEICESS protocols, only data of EICESS 1992 patients who were alive at a median time of 7.5 months after diagnosis, which equals the median time from diagnosis to first high-dose chemotherapy treatment of MetaEICESS 2007 and 1992 groups, were included.

### MetaEICESS 1992

11 patients were diagnosed with advanced Ewing sarcoma between 1992 and 2000. Diagnoses based upon histopathology. Median age at diagnosis that prompted MetaEICESS admission was 16 years (range 6–32 years). These patients were previously described [4]. Median time from diagnosis to first high-dose chemotherapy treatment was 8 months. 3 out of 11 had BM involvement at initial diagnosis (Supplementary Tables S1 and S2). Patients also received high-dose chemotherapy etc. irrespective of remission.

### MetaEICESS 2007

Diagnoses were made between 2007 and 2013 based upon histopathology and detection of translocations. Median age at diagnosis that prompted MetaEICESS admission was 15 years (range 8-17 years). Median time from diagnosis to first high-dose chemotherapy treatment was 5 months. 3 out of 7 patients had BM involvement at initial diagnosis (Supplementary Tables S1 and S2). Patients received high-dose chemotherapy and ensuing treatments irrespective of remission status.

### Staging

For MetaEICESS 1992 and MetaEICESS 2007 patients, stage and extent of disease were evaluated by cytological examination of BM aspirates, technetium scintigraphy, chest computed tomography positron emission tomography (PET) scans and whole-body MRI [4], which we introduced in 2003 for advanced Ewing sarcoma [6]. For whole body-MRI, short tau (time) inversion recovery and T1-weighted coronal imaging was applied in all patients. Lung disease was assessed by thoracic computed tomography. In EICESS 1992 patients, extent and stage of disease were evaluated by MRI, chest CT, BM aspirates and technetium scintigraphy bone scans.

### Induction chemotherapy and local treatment

All MetaEICESS patients received induction chemotherapy consisting of four blocks of VIDE (vincristine, ifosfamide, doxorubicin and etoposide) and two blocks of VIE when patients received primary tumor or metastases irradiation.

**Figure 1:**
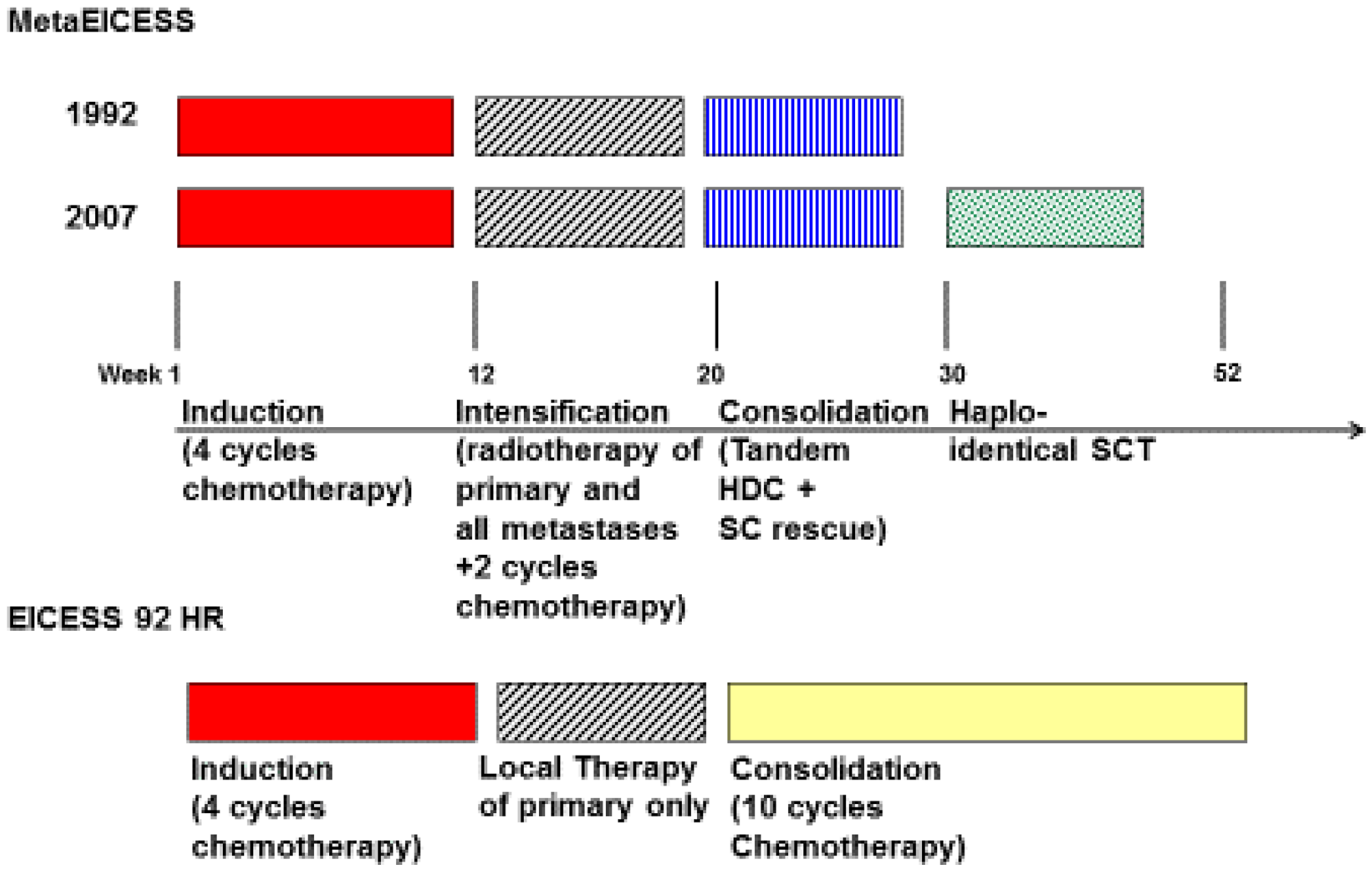
Schematic overview: Treatment strategies for MetaEICESS protocols 1992 and 2007 as well as EICESS 92 High-Risk (HR)

Autologous stem cell apheresis was conducted after the last two VIDE blocks. Involved lesion irradiation was delivered to the primary tumor (total dose 50-60 Gy), to the lungs (15-18 Gy), and to lymph node as well as multiple osseous metastases (45-50 Gy) in individual combinations. One patient received proton radiation (patient #1), all others received photon therapy. Radio-chemotherapy was followed by autologous stem cell transplantation (Figure 1).

In EICESS 1992 20/26 patients received only EVAIA and 6/26 patients received VAIA followed by EVAIA. As local therapy, 7/26 patients received surgery, 18/26 patients received radiation and 6/26 patients received both surgery and irradiation to the primary tumor site, whereas 1/26 patients did not receive local therapy. The total irradiation dose was 40–54 Gy with a general bone dose of 54 Gy, except for 40 Gy to the spine if more than three vertebrae were involved.

### Myeloablation and autologous stem cell transplantation

16/18 MetaEICESS patients received TandemME (MetaEICESS 1992) or ME/TopoTreo (MetaEICESS 2007) and 2 (all MetaEICESS 1992) patients HyperME myeloablation as previously described (Figure 1) [4, 6]. In MetaEICESS 2007 myeloablation consisted of melphalan (30 mg/m^2^/day on day −7 and −4 before first autologous stem cell transplantation) in combination with etoposide (1800 mg/m^2^/day on day −2 before first autologous stem cell transplantation) followed by application of topotecan (2 mg/m^2^/day on day −7 and −3 before second autologous stem cell transplantation) in combination with treosulfan (10 mg/m^2^/day on day −5 and −3 before second autologous stem cell transplantation). EICESS 1992 patients did not receive myeloablative therapy.

### Reduced intensity conditioning and allogeneic stem cell transplantation (MetaEICESS 2007)

After tandem high-dose chemotherapy with autologous stem cell transplantation, all MetaEICESS 2007 patients received reduced intensity chemotherapy followed by allogeneic stem cell transplantation with G-CSF mobilized peripheral blood stem cell products from family donors (Figure 1). Reduced intensity chemotherapy regimen consisted of fludarabine (30 mg/m^2^/day on day −9 and −5 before allogeneic stem cell transplantation) in combination with thiotepa (10 mg/m^2^/day on day −4 before allogeneic stem cell transplantation) and melphalan (35 mg/m^2^/day on day −3 and −2 before allogeneic stem cell transplantation). Seven patients received haploidentical grafts, one patient both a fully matched and an HLA 9/10 matched graft. Six patients were allo-transplanted in complete remission, whereas one patient was transplanted with residual disease (Supplementary Table S1).

### Statistical analysis

Statistical differences in were determined using the Graphpad Prism software, version 5.0 as well as R 2.11.0 (The R Foundation for Statistical Computing, Vienna Austria). In univariate analyses, statistical differences in disease-free survival were determined with the Kaplan–Meier method using the log rank (Mantel-Cox) test and the Breslow-Wilcoxon test. In the multivariate analysis, considered independent variables were patient age at diagnosis, gender, protocol (EICESS92 vs MetaEICESS) and BM involvement at diagnosis. Statistical differences were determined using the Wald test. Hazard ratios (HR), standard errors and confidence intervals (CI) are given when appropriate. In order to determine a correlation between BM involvement with the number of bone metastases at diagnosis the two-tailed t-test was used. In all tests p**<**0.05 was considered statistically significant. Final data base update was conducted on March 1st 2015.

## RESULTS

### Engraftment and GvHD (MetaEICESS 2007)

In MetaEICESS 2007 6/7 patients engrafted successfully after first transplant. Patient #5 had to be re-transplanted due to rejection. Three patients developed acute GvHD and one patient developed extensive chronic GvHD; Patient #5 developed BK virus induced hemorrhagic cystitis after allogeneic stem cell transplantation and was successfully treated with third party MSC. Two patients suffered adenovirus reactivation.

### Disease-free survival

In the MetaEICESS 2007 group median disease-free survival was was 28 months (range 11-73). 4/7 (0.57) MetaEICESS 2007 patients were alive in complete remission at a median of 34 months (range 11-88) after diagnosis that prompted MetaEICESS submission. 6/7 patients were in complete remission before allogeneic stem cell transplantation. One patient had progressive disease at the time of allogeneic stem cell transplantation. Median disease-survival after allogeneic stem cell transplantation was 21 months (range 0-64) (Supplementary Figure S1, Supplementary Table S2). Of these, three patients are alive in complete remission for an observation time of over 24 months. 2/6 patients, who were in complete remission at transplant relapsed after allogeneic stem cell transplantation. Patients #1 and #2 with relapse after allogeneic stem cell transplantation received various rescue therapies and survived for 11 months and 33 months after relapse, respectively (Supplementary Table S2).

In the MetaEICESS 1992 group, 3/11 (0.28) patients were alive at the end of the follow-up period, while 8/11 had died; 3/11 succumbed to treatment related complications and 5/11 to the underlying disease. Of those patients who had succumbed to complications, patient #10 died of treatment-related bacterial sepsis whereas patients #15 and #16 died of treatment-related secondary malignancies (liposarcoma and myelodysplastic syndrome) (Supplementary Table S2). Both latter patients were treated with allogeneic stem cell transplantation as rescue therapy *after* diagnosis of treatment related secondary malignancies. Median disease-free survival was 40 months (range: 6 months–20 years).

**Figure 2:**
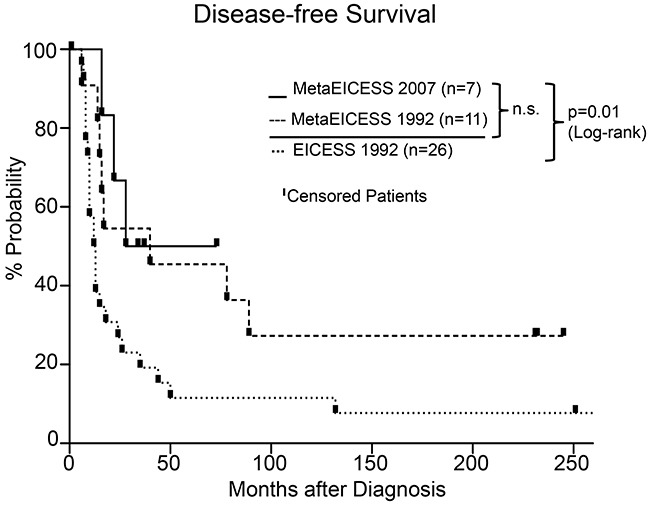
Disease-free survival in MetaEICESS 2007 (n=7) versus MetaEICESS 1992 (n=11) versus EICESS 1992 (n=26) from the date of diagnosis There was no statistically significant difference (n.s.) between the MetaEICESS 2007 and the MetaEICESS 1992 group. The EICESS 1992 survival curve differed significantly from the MetaEICESS 1992 and 2007 survival curves (Log-rank test, p=0.01; Breslow-Wilcoxon test p<0.01; HR 0.41; CI 0.21 to 0.82).

Death of disease and death of complication rates in the MetaEICESS 2007 vs. MetaEICESS 1992 group were 0.42 vs 0.45 and 0 vs 0.27, respectively. There was no statistical disease-free survival difference between both groups (Supplementary Table S2, Figure 2), however death of complications was lower in MetaEICESS 2007, i.e. *with* allogeneic stem cell transplantation. Karnofsky-Index was >90 in all survivors.

In the EICESS 1992 group 2/26 patients, 0.08 remained alive in complete remission at the end of the follow-up period. 24 patients had died, 22/24 of disease, 2/24 of complications. One of the latter two patients with treatment related complications died of secondary malignancy. 6/26 patients had BM involvement at diagnosis, all of whom died of disease. Median disease-free survival was 12.5 months (range 6 months–20 years).

Disease-free survival with the MetaEICESS 1992 and 2007 protocols was significantly better than with the EICESS 1992 protocol (Log-rank test, p=0.01; Breslow-Wilcoxon test p<0.01; HR 0.41; CI 0.21 to 0.82; Figure 2).

### Bone marrow involvement

**Figure 3:**
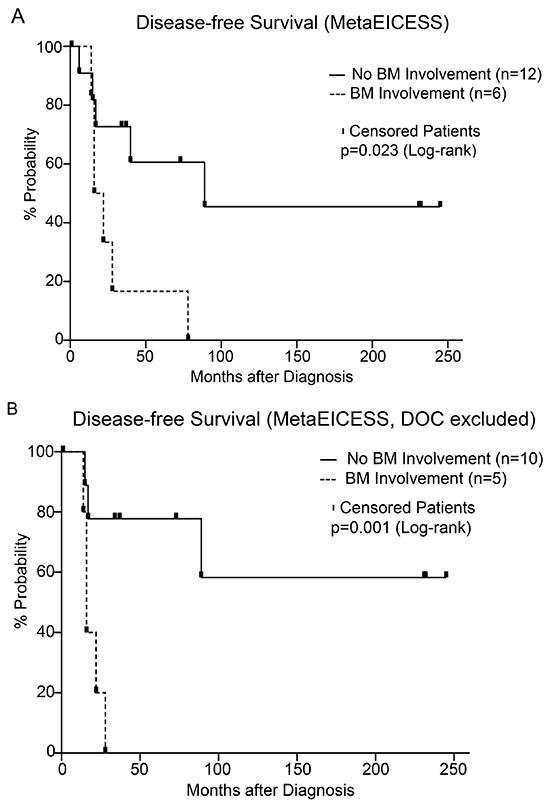
**A.** Disease-free survival with BM (n=6) versus without BM involvement (n=12) at diagnosis (MetaEICESS 1992 and 2007; p=0.023; HR 5.1; CI 1.2 to 20.8; Breslow-Wilcoxon test not significant). **B.** Poor disease-free survival with BM (n=5) versus without BM involvement (n=10) after exclusion of patients succumbing to treatment related complications (Log-rank test p<0.01; Breslow-Wilcoxon test p=0.013; HR 11.3; CI 2.0 to 63.3). n.s.; not significant.

3/7 patients in the MetaEICESS 2007 group and 3/11 in the MetaEICESS 1992 group had BM involvement at diagnosis. In all MetaEICESS patients 0/6 patients with BM involvement survived in contrast to 7/12 without BM involvement. All MetaEICESS patients with initial BM involvement (6/18) died: 3/3 MetaEICESS 2007 as well as 2/3 MetaEICESS 1992 patients with BM involvement died of disease; the third patient with BM involvement in MetaEICESS 1992 died of a secondary malignancy [4, 23]. The difference in disease-free survival between BM involvement vs. no BM involvement among MetaEICESS patients was statistically significant using the Log-rank test (p=0.023; HR 5.1; CI 1.2 to 20.8) but was insignificant using the Breslow-Wilcoxon test (Figure 3A). After exclusion of patients succumbing to treatment related complications (n=3), 5/5 patients with BM involvement in contrast to 2/10 patients without BM involvement had died of disease, leaving 7/10 patients without BM involvement in contrast to 0/5 with BM involvement in complete remission. The difference was statistically significant (Log-rank test p<0.01; Breslow-Wilcoxon test p=0.013; HR 11.3; CI 2.0 to 63.3; Figure 3B). In the EICESS 1992 group 6/26 patients had BM involvement at diagnosis: all of whom died of disease. When patients with bone metastases were excluded, survival fractions were 1.0 for MetaEICESS 2007 (4/4), 0.38 for MetaEICESS 1992 (3/8) and 0.2 (2/20) for EICESS 1992 patients. After exclusion of patients with bone marrow involvement the difference between both MetaEICESS protocols was not significant, whereas the difference between MetaEICESS versus EICESS 1992 remained significant (Log-rank p=0.01; Breslow-Wilcoxon p<0.01). In the whole study population of 44 patients there was no significant correlation between BM involvement and the number of metastases at diagnosis (two-tailed t-test p>0.05).

### Multivariate analyses

Upon multivariate analysis, neither age at diagnosis nor gender had an influence on disease-free survival. EICESS 1992 treatment as well as BM involvement at diagnosis was confirmed as risk factors for poor outcome (both p<0.01; Table 1). When MetaEICESS patients were excluded, multivariate analysis within the EICESS 1992 group confirmed BM involvement as a risk factor for poor outcome (Wald test p=0.03, Supplementary Table S3).

**Table 1:** Multivariate analysis (EICESS1992 and MetaEICESS; n=44); Poor disease-free survival in patients with EICESS 1992 treatment and/or BM involvement at diagnosis (both p<0.01*Wald-Test)

				HR	SE	95% CI	*P-value
**Disease-free Survival (n=44)**	**Age at Diagnosis**			0.95	0.03	0.99 to 1.11	**0.09**
	**Gender**	***male***	*Reference*				**0.06**
		***female***		1.84	0.33	0.29 to 1.03	
	**Protocol**	***EICESS 1992***	*Reference*				**<0.01**
		***MetaEICESS***		0.39	0.35	0.19 to 0.77	
	**BM Involvement at Diagnosis**	***Yes***	*Reference*				**<0.01**
		***No***		0.33	0.35	1.51 to 6.02	

Abbreviations: BM, bone marrow; HR, Hazard Ratio; SE, Standard Error; CI, Confidence Interval

## DISCUSSION

Our report deals with a new treatment protocol designed for a subgroup of patients with a rare disease with a hitherto dismal prognosis. Herein, we report on the results of our prospective single-center MetaEICESS 1992 and 2007 approaches in comparison to EICESS 1992. Primary and secondary endpoints were disease-free survival and toxicity. Even for the small numbers accumulated in this report (n=44), we had to compare patients treated within a time period of 22 years. This long period may compromise death of complications comparability between groups, e.g. due to improvement in supportive therapy or staging drift e.g. by introduction of PET-CT or possibly the extent of BM sampling. However, our studies continuously employed ≥ 2 metastatic bones as assessed by technetium scintigraphy as the entry criterion to the study and further evaluation by whole-body MRI. To the best of our knowledge, there is no published evidence that PET-CT is more sensitive than the combination of whole-body MRI, PET and BM aspirate in detecting additional bone metastases or BM involvement. Moreover, PET-CT did not affect the entry to our protocol. Our studies provide a unique long term evaluation of a very rare entity whose prognosis remained unchanged in most studies with reliable and sensitive diagnostic criteria conserved over this long term period: whole-body MRI and BM cytology.

A first finding of potential interest was that allogeneic stem cell transplantation does not increase death of complications. This finding contrasts with our previous finding of higher death of complications rates after this therapy modality [14]. This difference may be due to different conditioning.

In landmark as well as in recent studies, improvement of local therapy has proven crucial in the treatment of advanced Ewing sarcoma patients [11, 20, 24], whereas the role of high-dose chemotherapy regimen with autologous stem cell transplantation in the treatment of advanced Ewing sarcoma patients remains subject to an ongoing discussion [4, 7, 10, 15–17, 25–35]. The conflicting results of various studies including our previous analyses [6, 20] in contrast to the results of Meyers et al. [21] may be due to variation in intensity of local therapy. We cannot rule out that better results in MetaEICESS vs. EICESS are impacted by variation in induction chemotherapy. However this possibility does not compromise our conclusions that the MetaEICESS regimes in total yield a superior result as compared to EICESS.

Assessing the role of allogeneic stem cell transplantation it turned out that despite better toxicity control in MetaEICESS 2007 compared to MetaEICESS 1992, disease-free survival did not differ. This may be due to the higher incidence of BM involvement as a confounding variable in MetaEICESS 2007 3/7 (0.43) vs. 3/11 (0.27) conferring poor prognosis. Thus, MetaEICESS protocols yield long-term survival in advanced Ewing sarcoma patients but do not eliminate the negative impact on disease-free survival of BM involvement. In a recent study, 87 out of 507 (0.17) ES patients with disseminated disease showed BM involvement [30]. While our previous observation that the presence of multiple bone or BM metastases is associated with poor prognosis in ES has been confirmed in this study, the single contribution of multiple bone metastases vs. BM involvement has not been assessed so far. In our study population of 44 patients there was no significant correlation between BM involvement and the number of bone metastases at diagnosis. Of note, Kopp et al. reported recently that BM involvement correlates with the number of bone metastases [3].

Given the albeit improved but still unsatisfactory results with high dose consolidation necessitating (autologous) rescue we attempted to assess the curative role of haploidentical allogeneic stem cell transplantation in MetaEICESS 2007. Following the prospective analysis we retrospectively discovered BM involvement as a new distinct and independent risk factor beyond multiple bone metastases. The cut-off for multiple bone metastases used here was ≥2. Thus, BM involvement may be indicative of a higher number of multiple bone metastases.

Since the incidence of BM involvement turned out to be higher in MetaEICESS 2007 vs. MetaEICESS 1992, we were not able to clearly assess the potential of allogeneic stem cell transplantation to improve the prognosis of patients with multiple bone metastases but no BM involvement; this remains to be defined. Of note, results of MetaEICESS 2007 were not inferior to MetaEICESS 1992 despite the higher incidence of BM involvement in MetaEICESS 2007. Moreover, results of MetaEICESS 2007 were superior to EICESS92 despite the higher incidence of BM involvement in MetaEICESS 2007.

So far, BM involvement has not been published as a distinct risk factor beyond multiple bone metastases in advanced Ewing sarcoma. In addition to variation in local therapy, the unknown distribution of the risk factor BM involvement may explain, at least in part, the afore mentioned conflicting results of previous studies e.g. Meyers et al. [21] vs. Burdach et al. [6, 20], Barker et al. [36] and Rasper et al. [7].

Taken together, BM involvement identifies a subgroup of advanced Ewing sarcoma patients with fatal outcome irrespective of therapy whereas advanced Ewing sarcoma patients with multiple bone metastases but without BM involvement may be cured with the MetaEICESS protocols. Allogeneic stem cell transplantation yielded no deaths of complications in this setting and quality of life was good in all survivors in comparison to former analyses [14, 37]. Despite the fact that eligibility criteria were identical, treatment groups were not randomized and not treated contemporaneously. Thus, there may be potential bias in eligibility that may impact the observation that MetaEICESS may be associated with improved outcome. Nevertheless, allogeneic stem cell transplantation warrants future evaluation to improve prognosis in particular of advanced Ewing sarcoma patients without BM involvement, whereas patients with BM involvement require additional novel approaches.

## SUPPLEMENTARY FIGURE AND TABLES






