# Supplementary material for: Bone marrow involvement identifies a subgroup of advanced Ewing sarcoma patients with fatal outcome irrespective of therapy in contrast to curable patients with multiple bone metastases but unaffected marrow
Source: Oncotarget. 2016 Jul 29;7(43):70959–68. doi: 10.18632/oncotarget.10938 (PMC5342601; doi:10.18632/oncotarget.10938)
Supplement: Supplementary file 1 [file oncotarget-07-70959-s001.pdf]

Bone marrow involvement identifies a subgroup of advanced Ewing sarcoma patients with fatal outcome irrespective of therapy in contrast to curable patients with multiple bone metastases but unaffected marrow

SUPPLEMENTARY FIGURE AND TABLES

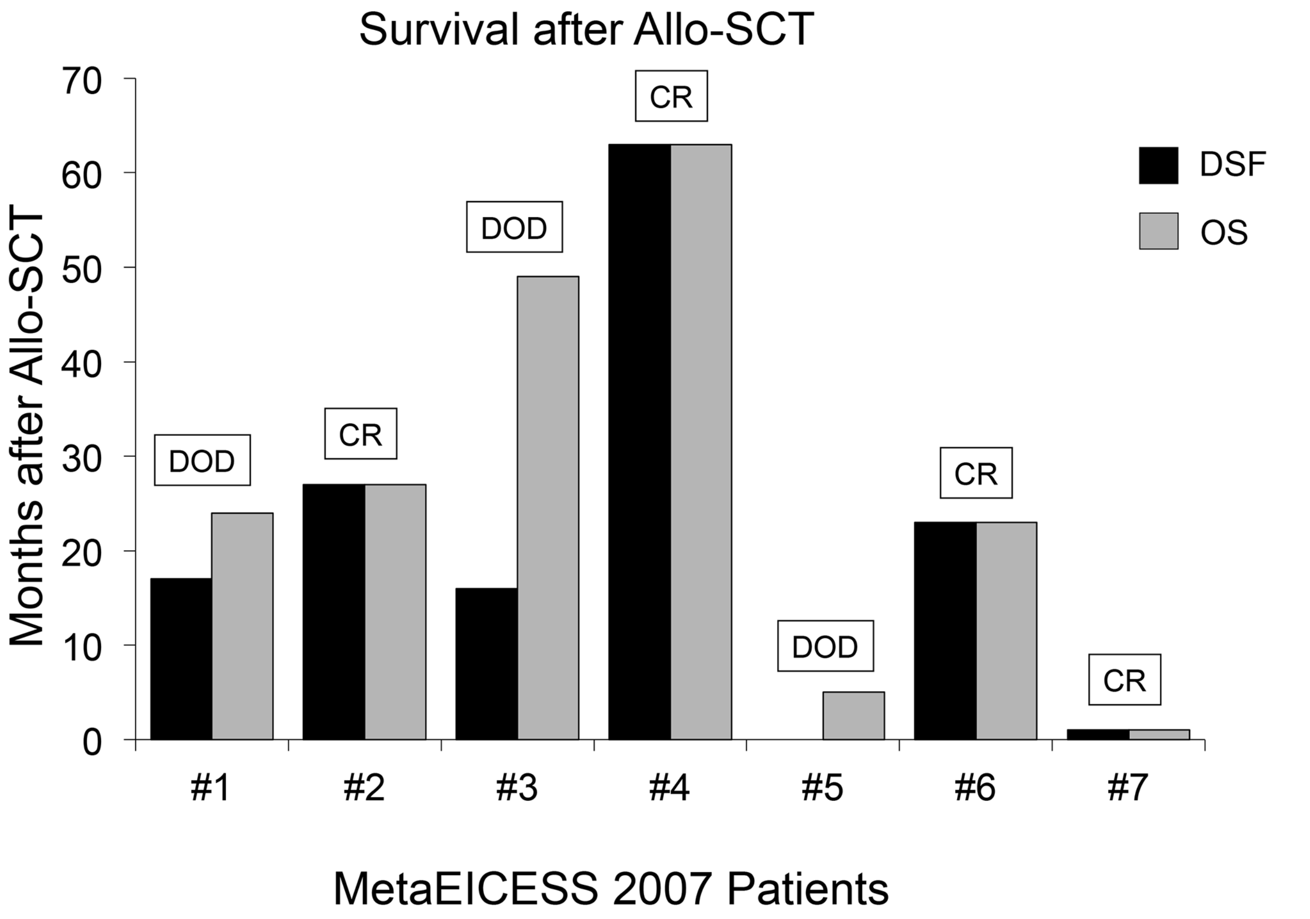

Supplementary Figure S1: Individual survival after allogeneic stem cell transplantation. Abbreviations: allogeneic stem cell transplantation, allogeneic stem cell transplantation; DOD, death of disease; CR complete remission; DFS, disease-free survival; OS, overall survival after allogeneic stem cell transplantation.

Supplementary Table S1: Individual patient characteristics and treatment.

See Supplementary File 1

Supplementary Table S2: Individual outcomes.

See Supplementary File 2

Supplementary Table S3: Multivariate analysis (only EICESS 1992; n=26); Confirmation of poor disease-free survival within EICESS 1992 patients with BM involvement at diagnosis (p=0.03 *Wald-Test)

				HR	SE	95% CI	**P*-value
**Disease-free Survival (EICESS1992)**	**Age at Diagnosis**			0.97	0.03	0.97 to 1.10	**0.37**
	**Gender**	***male***	*Reference*				**0.06**
		female		1.94	0.38	0.98 to 4.36	
	**BM Involvement at Diagnosis**	***Yes***	*Reference*				**0.03**
		No		0.38	0.44	1.10 to 6.18	

Abbreviations: BM, bone marrow; HR, Hazard Ratio; SE, Standard Error; CI, Confidence Interval.
